# Spiritual Support Among African American and Caucasian ADRD Caregivers: A Risk and Resilience Study

**DOI:** 10.1093/geroni/igab046.031

**Published:** 2021-12-17

**Authors:** Scott Wilks, Wanda Spurlock, Sandra Brown, Jennifer Geiger, Sarah Choate, Katherine Kirsch, Alisha Thompson, Cassie Slaton

**Affiliations:** 1 Louisiana State University, Baton Rouge, Louisiana, United States; 2 Southern University College of Nursing and Allied health, Baton Rouge, Louisiana, United States; 3 East Tennessee State University Dept. of Social Work, Johnson City, Tennessee, United States; 4 Louisiana State University School of Social Work, Baton Rouge, Louisiana, United States

## Abstract

Research shows African Americans at greater risk of developing Alzheimer’s disease and related dementias (ADRD) compared to Caucasians, suggesting African American ADRD caregivers are rising in numbers at a greater rate than Caucasian counterparts. A recent study indicated spiritual wellbeing differences among these caregiver groups. Using a quasi-follow-up of members of a larger caregiver sample, the purpose of this study was to test spiritual support as a moderator via a risk-and-resilience framework. Secondary data analysis from a sample of 691 ADRD caregivers examined data on demographics and standardized measures of spiritual support, caregiver burden, and psychological resilience. One-third of the sample reported as African American. Resilience negatively regressed, though not significantly, on caregiving burden among both groups. Spiritual support positively, significantly impacted resilience among both groups, slightly stronger among African Americans. Spiritual support did not significantly moderate risk with either group. Implications for professional healthcare practice are discussed.

